# Neuron specific enolase is a potential target for regulating neuronal cell survival and death: implications in neurodegeneration and regeneration

**DOI:** 10.20517/2347-8659.2017.59

**Published:** 2017-12-06

**Authors:** Rachel Polcyn, Mollie Capone, Azim Hossain, Denise Matzelle, Naren L. Banik, Azizul Haque

**Affiliations:** 1 Department of Microbiology and Immunology, Hollings Cancer Center, Medical University of South Carolina, Charleston, SC 29425, USA; 2 Department of Neurosurgery, Medical University of South Carolina, Charleston, SC 29425, USA; 3 Ralph H. Johnson Veterans Administration Medical Center, Charleston, SC 29401, USA

Enolase is a multifunctional enzyme primarily involved in catalyzing the conversion of 2-phosphoglycerate to phosphoenolpyruvate during glycolysis and the reverse reaction during gluconeogenesis^[1–4]^. Though typically expressed in the cytosol, enolase has been shown to migrate to the cell surface upon inflammatory signal^[3]^. It then enhances antigen presentation for the invasion of host cells via plasminogen binding and subsequent plasmin activation, leading to degradation of the extracellular matrix. Cell surface expression of enolase, possibly due to an association with the urokinase-type plasminogen activator (uPA)/uPA receptor complex, additionally induces the production of reactive oxygen species, nitric oxide, and pro-inflammatory cytokines [tumor necrotic factor (TNF)-α, interleukin (IL)-1β, interferon-γ, and transforming growth factor-β] and chemokines [monocyte chemotactic protein 1 and macrophage inflammatory protein (MIP)-1α] to augment neurodegenerative response^[3,5]^. Lysosomal proteases, especially cathepsins (e.g. Cathepsin X or Cat X), are instrumental in processing several neuronal proteins that generate either harmful or neuroprotective functions. Cathepsins and a neutral protease, calpain, also have regulatory functions in antigen processing and presentation, thereby inducing immune responses that can have either detrimental or beneficial effects on neuronal cells. This editorial discusses the relationships between neuron specific enolase (NSE) and Cat X activity in neuronal cells with a special focus on their implications for neurodegeneration and neuroprotection.

Distinct dimeric isoforms of enolase are composed of non-covalently linked α, β, or γ subunits and are tissue-specific^[3]^. During development, injury, or disease, α-enolase (enolase 1, non-neuronal enolase, ENO1), which is primarily found in adult tissue, can be converted to γ-enolase (enolase 2, NSE) in neurons and neuroendocrine cells. Similarly, α-enolase is converted into β-enolase (enolase 3, muscle specific enolase, ENO3) in muscle tissue. NSE exists as both αγ and γγ isoforms in neurons, γγ in microglia and oligodendrocytes, and αγ in astrocytes^[3,4]^. Notably, enolase levels have been shown to be significantly increased in astrocytes and microglia following spinal cord injury (SCI), an observation indicating a possible role for NSE in different pathologies associated with the neuroinflammatory, apoptotic, and neuroprotective activity of these interacting glial cells^[6,7]^. Due to its specific location in neurons and neuroendocrine cells and its upregulated secretion following axonal damage, NSE has been implicated as a biomarker of functional damage to neurons in SCI and several other pathophysiological conditions: traumatic brain injury, stroke, ischemia-reperfusion injury, cardiac arrest, neuroblastoma, small-cell lung cancer, and Alzheimer’s disease (AD)^[2–5]^.

SCI is a debilitating neurological disorder that occurs in two main phases: primary and secondary injury^[3,8]^. The initial primary injury, resulting from laceration, contusion, compression, and/or contraction of the neural tissue, is the immediate, irreversible damage to the spinal cord and associated axons, cells, and blood vessels. However, a diverse array of secondary injury mechanisms, including hypoxia, ischemia, excitotoxicity, inflammation, and apoptosis, expands the injury site and impairs pro-survival activity following primary injury^[9]^. These secondary processes are reversible and as such have been a principle target of SCI treatment research ^[8]^. Substantial reduction in blood supply from primary injury triggers ischemia, oxidative stress, and microglial activation that drive the release of pro-inflammatory cytokines and chemokines at the injury site. Under the hypoxic-ischemic conditions immediately following SCI, an influx of Ca^2+^ results in activation of calpain, caspase, and phospholipases^[10,11]^. Calpain then degrades cytoskeletal proteins and leads to apoptosis. Additional neuronal death can be attributed to glutamate excitotoxicity following the injury^[9]^. Though these effects are primarily neurodegenerative, macrophages may function as pro- or anti-inflammatory agents in SCI, depending on the M1/M2 macrophage cell ratio in the injured microenvironment.

Our group has shown that SCI treatment in a male Sprague-Dawley rat model using ENOblock, a small molecule inhibitor of enolase, corresponded to a reduction of NSE expression in SCI tissues and a significant decrease in serum NSE, matrix metallopeptidase (MMP)-9 protein expression in injured tissue, serum pro-inflammatory cytokines (TNF-α, IL-1β, and IL-6) and chemokines (MIP-1α and IP-10), and glial activation ^[2]^. Elevated MMP-9 expression can promote the activation of microglia and astrocytes, leading to the release of inflammatory cytokines and chemokines that promote cell death. Expression of these pro-inflammatory cytokines (TNF-α, IL-1β, and IL-6) is known to induce hyperalgesia, allodynia, and apoptosis of neuronal and glial cells in association with the secondary damages of SCI^[2,12–15]^. Additionally, MIP-1α has been shown to mediate microglia accumulation and neuroinflammation in brain injury ^[16]^. IP-10, expressed by astrocytes in response to N-methyl-D-aspartate-dependent excitotoxicity, activates the mitogen-activated protein kinase (MAPK)/extracellular signal-regulated kinase (ERK) pathway in mouse cortical neurons, indicating a role for this chemokine in mediating cell proliferation and apoptosis under neurodegenerative conditions^[17,18]^. The reduction of MMP-9 and the aforementioned pro-inflammatory cytokines and chemokines by ENOblock indicates the potential for this treatment in attenuating neural damages associated with inflammatory response during secondary injury mechanisms of SCI.

While previous research on the role of NSE in secondary injury mechanisms of SCI has focused on neurodegenerative effects, namely the association between the overexpression of NSE and an inflammatory cascade leading to neuronal cell death, future studies should additionally investigate the role of NSE in pro-survival and regeneration activity via cellular signaling pathways in acute and chronic SCI. NSE has been shown to exhibit neurotrophic activity in controlling neuronal survival, differentiation, and neurite regeneration of human neuroblastoma SH-SY5Y cells via activation of the phosphoinositide 3-kinase (PI3K)/Akt and MAPK/ERK signaling pathways, which regulate cytoskeleton reorganization and transcriptional factor activation in promotion of cell survival and neurite outgrowth^[1]^. A neuroprotective effect of NSE expression and activity was also observed in a mouse model of AD against amyloid-β-related neurodegeneration^[5]^. Additionally, the neuritogenic activity of NSE is associated with RhoA kinase inactivation within the PI3K/Akt pathway and affects neurite outgrowth through rapid actin polymerization^[1]^. NSE likely exhibits similar neuroprotective activity for cell survival, differentiation, and migration following SCI and other neurodegenerative conditions via the PI3K/Akt and MAPK/ERK pathways. Further research evaluating the interaction between NSE and these pathways in SCI secondary injury mechanisms for more clarification would be interesting.

Investigation into the neurotrophic activity of NSE in a mouse model of the neurodegenerative condition AD has shown that NSE can be regulated by Cat X, a lysosomal cysteine protease that cleaves the C-terminal end of the NSE enzyme under acidic conditions^[5]^. The C-terminal peptide of NSE, which is not involved in plasminogen binding (due to the absence of lysine) or glycolytic function, contains a PDZ-binding domain for the scaffold protein γ-1 syntrophin that enables NSE to relocate to the plasma membrane via actin filament, as evidenced by their co-localization^[4]^. This C-terminal peptide has been shown to have a pro-survival effect on PC12 cells^[19]^. The cleavage of NSE at this site by Cat X severely affects its ability to function in neuronal cell differentiation for pro-survival activity or cell death. Because of the known involvement of PI3K/Akt and MAPK/ERK signaling pathways in the activation of cathepsin B, a similar cysteine protease, in glioma, Cat X activity is likely associated with these same pathways^[20]^.

An additional cysteine protease, calpain, is involved in the neuroinflammatory response to SCI^[21]^. Calpain is found in the cytosol and is active under neutral pH conditions upon Ca^2+^ activation. The role of calpain in apoptosis has been clearly demonstrated, and its activation in SCI conditions has been shown to lead to cytoskeletal and myelin protein cleavage. Calpeptin, a calpain inhibitor, can exhibit neuroprotective effects against excitotoxic apoptosis, reducing neuronal cell death^[22]^. While inhibition of enolase by ENOblock alters cellular growth, cytokines/chemokines, and inflammatory markers^[2,23]^, it is unknown if ENOblock acts on Cat X and regulates its function. Our group has found increased calpain activity and cell-specific overexpression in astrocytes, microglia, macrophages and T cells in inflammatory demyelinating diseases^[24–26]^. However, the effects of calpain inhibition (calpeptin) on NSE and Cat X functions remain to be investigated. Calpeptin, which is a cysteine protease inhibitor, could possibly target Cat X, leading to inhibition of NSE-mediated inflammatory events and promotion of neuronal cell survival. Since NSE is a substrate of Cat X, evaluating both ENOblock and calpeptin as potential mediators of NSE expression and activity in neuronal cells following SCI and other neurological disorders.

At certain levels, NSE can support regeneration of neuronal cells^[1,5]^. NSE-mediated activation of the PI3K/Akt and MAPK/ERK pathways likely supports cell survival and regeneration. On the other hand, these pathways also likely activate Cat X, an enzyme that cleaves NSE. Cat X activity would likely result in a reduction of NSE-mediated activation of PI3K/Akt and MAPK/ERK pathways and result in cell death. It would be interesting to investigate the role of ENOblock in regulating PI3K/Akt and MAPK/ERK pathways and Cat X activity. Future studies to elucidate the role of PI3K/Akt and MAPK/ERK pathways in defining cell fate are warranted. Mediation of the Cat X activity in association with these pathways could result in partial as opposed to total degradation of NSE, thus reducing NSE and Cat X mediated cell death and providing a promising future therapeutic target for reversal of secondary injury mechanisms in acute and chronic SCI as well as other neurological conditions.

In conclusion, several future avenues for research on the mechanisms of NSE expression and activity in neurons and glia and the process of neurodegeneration and regeneration following neurological impairment have been discussed. NSE, once migrated to the plasma membrane, takes part in cellular activation, production of inflammatory cytokines and chemokines, and induction of neuronal cell death (neurodegeneration)^[1,3]^. The regulated expression of NSE may promote neuronal survival (neuroprotection or regeneration) via cell survival pathways. Previous research has focused on the harmful effects of NSE overexpression following neuronal damage. However, future studies should address the conditions leading to preferential differentiation into pro-survival activity or neuronal cell death and specific methods for regulating NSE and Cat X activity to mediate the secondary damages associated with SCI. The role of Cat X in secondary injury remains unknown, as does the influence of SCI on Cat X expression and activity. Additionally, the direct and indirect targets of ENOblock treatment have yet to be determined. Calpeptin, which acts on calpain to reduce neuronal cell death, may similarly act on Cat X to regulate NSE activity. The effects of these inhibitors on neurodegeneration and/or neuroprotection and their potential interaction with the PI3K/Akt and MAPK/ERK pathways remain to be determined. This editorial has highlighted several potential intermediary effectors associated with neurodegeneration and neuroprotection in SCI and other neuropathological conditions. Significant research is needed to further evaluate these possible mechanisms and their potential for translation into future preclinical and clinical treatments.
